# Event and Comment

**Published:** 1919-11

**Authors:** 


### DENTAL REGISTER

THE MAGAZINE OF DENTISTRY

Vol. LXXIII NOVEMBER, 1919 No. 11

### EVENT AND COMMENT

**The Annual Meeting of the National Dental Association**

The various national organizations held their annual meetings at New Orleans the week of October 20th with a good attendance, considering the distance to be traveled and the time of the year when the meetings were held. The weather, while not so hot as the Chicago week of last year, was extremely uncomfortable and much complaint was made on that score. The dentists and people of New Orleans did all that was expected of them to make the visitors comfortable and happy and no complaints are recorded against the hospitality extended. The programme of meetings was carried out as announced, and two half days were given over to clinics. The various societies and allied organizations held their meetings and as usual filled in all the available time, so that there was little time or opportunity for recreation for those who held offices or had committee work to do.

The new society of Prosthodontists had a good programme and completed its organization by adopting a constitution, etc. This society has undertaken a big task and will hold its meeting next summer in Minneapolis and spend a whole month in endeavoring to get its work fully organized.

The Educational Council seems to have made substantial progress in getting the educational bodies to harmonize some of their differences and it now seems probable that we may have a better understanding between the several colleges and state boards in regard to educational standards. If this can be accomplished on a proper basis it will be the greatest event of the year.

The Scientific Foundation and Research Commission had a rather strenuous session, which fortunately terminated in a happy manner, and the prospects for a much more active program of endeavor by this organization is promised in the near future. It is hoped that considerable increase in the financial resourses of the Research Commission will make possible increased grants for research in lines heretofore not undertaken.

The work of the National Association is so varied and complex, that it is practically impossible to crowd it all into a four or five-day session that any one person can attend, and consequently one always comes away from this meeting with the feeling of having neglected his opportunity to take advantage of all the good things offered. But the large membership of this body will make it impossible to carry on a satisfactory program of professional matters, because of the increasing pressure of executive details which consume so much of the officers" and visitors' time. It will always be a busy week unless some system of carrying on the executive matters is devised that will eliminate them very largely.

---

**A Free Dental Clinic in Every Public School** The First and Second District societies of New York have undertaken, through their Oral Hygiene committees, to carry out a campaign to create a public demand for a free dental clinic for every school in the city. This is to be done by a circular letter sent to the parents of the children, giving some of the facts as to nature and extent of dental diseases, and the detrimental influences on the health and mental development of the children because of the great prevalence of these diseases among school children.

In addition to this form letters are to be sent to the parents who are asked to sign them and mail them to the aldermen and city officials who have control of the school and health affairs of the city. Clergymen, school teachers and officers also are asked to write letters to the health officer and school boards calling attention to the importance of this matter.

The city officials are to be fully informed as to the economic value of such a proposition as well as its health significance. If this does not accomplish the object, the fault will not be because the city authorities are ignorant of the importance of such a provision for the good health of New York's children.

